# Posterolateral rotatory instability of elbow: An uncommon entity

**DOI:** 10.4103/0019-5413.41862

**Published:** 2008

**Authors:** Md. Quamar Azam, AA Iraqi, Anjum Syed, M Abbas

**Affiliations:** Department of Orthopedic Surgery, JN Medical College, Aligarh Muslim University, Aligarh - 202 002, Uttar Pradesh, India

**Keywords:** Elbow, ligamentous injury, posterolateral instability

## Abstract

Posterolateral rotatory instability of elbow is an exceedingly uncommon entity, which results from injury to the lateral ligamentous complex. Failure of adequate healing of lateral collateral ligaments may necessitate its surgical repair or reconstruction. We describe here a boy 12 years of age who was initially treated as soft tissue injury and later presented with instability of the same elbow. He later required repair of lateral ulnar collateral ligament.

## INTRODUCTION

The elbow is a highly constrained and stable joint. Stability depends on the bony architecture and integrity of ligaments, capsule and muscles around the joint. Recently, attention has focused on the lateral ulnar collateral ligament as the primary constraint to posterolateral instability. Recurrent dislocation of elbow, unlike shoulder is an exceedingly rare phenomenon. We describe here a case of posterolateral rotatory instability of elbow, the importance of clinical examination (posterolateral rotatory instability test [PLRI test]) despite previous normal investigation and the role of the lateral ligamentous complex.

## CASE HISTORY

**Figure 1:**
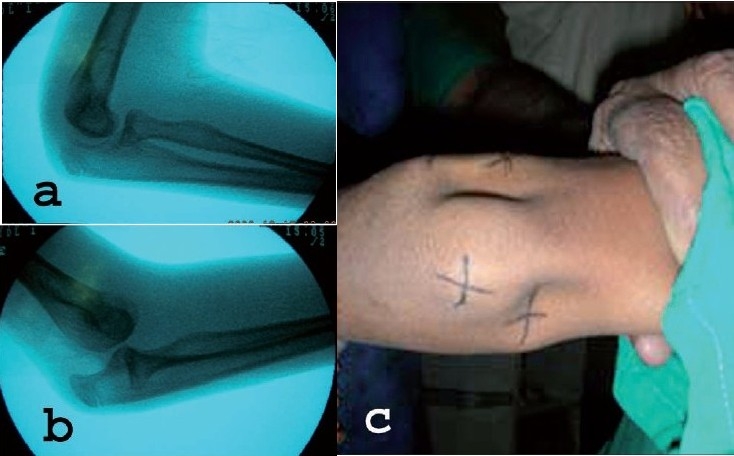
(a) Lateral view of elbow as seen under fluoroscope showing no abnormality. (b) Fluoroscopic image showing dislocated elbow after performing posterolateral instability test. (c) Clinical photograph of elbow showing appearance of a dimple over the radial head of the dislocated elbow

A 12-year-old boy presented with history of giving way of his right elbow for last one and half month. He had sustained an injury to the same elbow three months back. Diagnosis of soft tissue injury was made after getting a radiograph (anteroposterior and lateral view of the elbow) and above elbow slab was applied for three weeks. After removal of plaster, when physiotherapy was started the boy had a sense of instability while performing certain activities like trying to push some objects or while taking support to get up from chair. Over the last one and half months he noted that the elbow moved out of its position with an audible snapping sound [Figure 1a]. He had by now learnt the maneuver of bringing the elbow in normal position.

General examination revealed no ligamentous laxity. His previous radiographs reviewed were normal. Clinical examination was unremarkable except for PLRI test being positive. The test was performed without anesthesia under image. Holding the patient's forearm in full supination, the elbow was slowly flexed and the examiner applied a valgus force coupled with axial loading. At about 40-50° of flexion the elbow dislocated [Figure 1b] with a clunk. The dislocation was obvious with appearance of dimple proximal to the radial head [Figure 1c].

The patient was initially referred to a physiotherapist for elbow strengthening exercises but as the symptoms did not improve, surgery was performed.

**Operative procedure:** Repair of lateral ulnar collateral ligament was done through a posterior midline incision as described by Osborne and Cotterill.^1^ Under general anesthesia the patient was placed in lateral position. Through a posterior midline incision, Kocher's interval between extensor carpi ulnaris and anconeus was identified. The interval was developed to expose the lateral joint capsule, the lateral epicondyle and supinator crest of ulna. The bone of the lateral epicondyle was cleared of soft tissues. Using catgut suture, lateral ligamentous complex was reattached to the lateral epicondyle thereby achieving transosseus ligament repair. Above elbow slab was given for three weeks and then elbow was gradually mobilized. At the end of one year at the last follow-up, patient had stable elbow with full range of motion.

## DISCUSSION

Elbow is the most common joint dislocation in children^1^ and the second most common in adults (after shoulder). However, recurrent dislocation is an exceedingly rare phenomenon. Linscheid and Wheeler^2^ in a series of 110 elbow dislocations found only two cases of recurrent dislocation.

The humeroulnar joint is a hinge (ginglymus) joint with inherent stability provided by the depth of the trochlear fossa, further enhanced by the olecranon and coronoid process. Three primary stabilizers of the elbow are the humeroulnar articulation, the lateral collateral ligament complex and the medial collateral complex. Secondary stabilizers include radial head, the capsule and the common flexor and extensor origin. Over the period of a decade, attention has shifted from medial collateral ligaments (MCL) to lateral collateral ligaments (LCL) as the primary constraint to the posterolateral stability of the elbow.^3^–^5^

Morrey and An,^6^ recognized that the lateral ulnar collateral ligament (LUCL) is a thickening of the capsule that attaches proximally from the lateral humeral epicondyle to the tubercle of the supinator crest of the ulna. He further stressed that besides stabilizing the lateral aspect of the elbow, LUCL also acts as a posteror buttress for the radial head to prevent its subluxation. Posterolateral rotatory instability, a clinical syndrome, was first described by O'Driscoll *et al.*,^3^ who postulated that an insufficiency of the LUCL allows abnormal supination (external rotation) of the ulna on the humerus. The radial head, being locked in the sigmoid notch of the proximal ulna by annular ligament, subluxates posterior to the capitulum.

Mehta *et al.*,^7^ noted this abnormality to be usually post-traumatic and presented with locking, snapping, clicking, catching and recurrent dislocation of elbow. They used arthroscopy to classify patients with PLRI into three groups: (1) those with isolated PLRI are managed with lateral reconstruction; (2) those with associated abnormal medial joint space opening are managed with circumferential graft that reconstructs the MCL and LUCL; (3) patients with PLRI with associated arthritis. The last group requires assessment of instability and arthritis separately.

Osborne and Cotterill^1^ obtained excellent results after treating eight cases with transosseus repair. Nestor *et al.*,^8^ also had an opportunity to treat three such cases where they reported excellent outcome after LUCL repair. Hassman *et al.*,^9^ successfully treated four patients who had recurrent dislocation of elbow with Osborne and Cotterill repair of lateral ligament complex.

To summarize, PLRI of elbow is a rare clinical entity that often results due to either laxity or detachment of the ulnar part of the lateral collateral ligament. Clinical diagnosis is made by performing PLRI test as described by O'Driscoll. Plication of the ligament, reattachment or both restores the functional integrity of the elbow.
